# Supplementary material for: The composition of British bird communities is associated with long-term garden bird feeding
Source: Nat Commun. 2019 May 21;10:2088. doi: 10.1038/s41467-019-10111-5 (PMC6529538; doi:10.1038/s41467-019-10111-5)
Supplement: Supplementary file 1 — Supplementary Information [file 41467_2019_10111_MOESM1_ESM.pdf]

Supplementary Information:

The composition of British bird communities is associated with long-term garden bird feeding

Plummer et al.

Supplementary Table 1| Bird food types advertised in *Birds* magazine between 1973 and 2005.

Broad category	Food type	First year advertised
Nuts	Peanuts	1973
	Other single nuts	2000
Seeds	Sunflower seeds	1973
	Black sunflower seeds	1991
	Sunflower hearts	1998
	Niger seed	1998
	Other single seeds	1988
Grains	Oats	1973
	Other single grains	1973
Fruits	Dried fruit	2000
Live foods	Live foods	1994
Fats	Fat balls	1997
	Suet cake	1998
	Suet pellets	2000
Seed and/or grain mixes	Seed mix	1993
	Grain mix	1973
	Seed and grain mix	1973
	Insectivorous mix	1973
	Multi-species mix	1997
	High energy mix	1998
	Finch mix	2001

Supplementary Table 2| All species recorded using garden bird feeders in winter between 1973/74 and 2012/13 within the Garden Bird Feeding Survey (GBFS). Species defined as feeder-users are in bold.

Species	Species	Species
Mute swan (*Cygnus olor*)	Ring-necked parakeet (*Psittacula krameri*)	Ring ouzel (*Turdus torquatus*)
Canada goose (*Branta canadensis*)	Barn owl (*Tyto alba*)	**Blackbird (*Turdus merula*)**
Shelduck (*Tadorna tadorna*)	Little owl (*Athene noctua*)	**Fieldfare (*Turdus pilaris*)**
Mandarin duck (*Aix galericulata*)	Tawny owl (*Strix aluco*)	**Song thrush (*Turdus philomelos*)**
Teal (*Anas crecca*)	Short-eared owl (*Asio flammeus*)	**Redwing (*Turdus iliacus*)**
Mallard (*Anas platyrhynchos*)	Kingfisher (*Alcedo atthis*)	**Mistle thrush (*Turdus viscivorus*)**
Pochard (*Aythya ferina*)	Hoopoe (*Upupa epops*)	Spotted flycatcher (*Muscicapa striata*)
Red-legged partridge (*Alectoris rufa*)	Wryneck (*Jynx torquilla*)	**Robin (*Erithacus rubecula*)**
Quail (*Coturnix coturnix*)	Green woodpecker (*Picus viridis*)	Pied flycatcher (*Ficedula hypoleuca*)
**Pheasant (*Phasianus colchicus*)**	**Great spotted woodpecker (*Dendrocopos major*)**	Black redstart (*Phoenicurus ochruros*)
Golden pheasant (*Chrysolophus pictus*)	Lesser spotted woodpecker (*Dendrocopos minor*)	Whinchat (*Saxicola rubetra*)
Lady Amherst's pheasant (*Chrysolophus amherstiae*)	Great grey shrike (*Lanius excubitor*)	Stonechat (*Saxicola rubicola*)
Little egret (*Egretta garzetta*)	Chough (*Pyrrhocorax pyrrhocorax*)	Wheatear (*Oenanthe oenanthe*)
Grey heron (*Ardea cinerea*)	**Magpie (*Pica pica*)**	**Dunnock (*Prunella modularis*)**
Red kite (*Milvus milvus*)	**Jay (*Garrulus glandarius*)**	**House sparrow (*Passer domesticus*)**
Hen harrier (*Circus cyaneus*)	**Jackdaw (*Corvus monedula*)**	**Tree sparrow (*Passer montanus*)**
Goshawk (*Accipiter gentilis*)	**Rook (*Corvus frugilegus*)**	Yellow wagtail (*Motacilla flava*)
**Sparrowhawk (*Accipiter nisus*)**	**Carrion crow (*Corvus corone*)**	Grey wagtail (*Motacilla cinerea*)
Buzzard (*Buteo buteo*)	Hooded crow (*Corvus cornix*)	**Pied wagtail (*Motacilla alba*)**
Kestrel (*Falco tinnunculus*)	Raven (*Corvus corax*)	Richard's pipit (*Anthus richardi*)
Merlin (*Falco columbarius*)	**Goldcrest (*Regulus regulus*)**	Tree pipit (*Anthus trivialis*)
Peregrine (*Falco peregrinus*)	Firecrest (*Regulus ignicapilla*)	Meadow pipit (*Anthus pratensis*)
Water rail (*Rallus aquaticus*)	**Blue tit (*Cyanistes caeruleus*)**	Rock pipit (*Anthus petrosus*)
Moorhen (*Gallinula chloropus*)	**Great tit (*Parus major*)**	**Chaffinch (*Fringilla coelebs*)**
Coot (*Fulica atra*)	Crested tit (*Lophophanes cristatus*)	**Brambling (*Fringilla montifringilla*)**
Oystercatcher (*Haematopus ostralegus*)	**Coal tit (*Periparus ater*)**	**Greenfinch (*Chloris chloris*)**
Lapwing (*Vanellus vanellus*)	**Marsh tit (*Poecile palustris*)**	Serin (*Serinus serinus*)
Jack snipe (*Lymnocryptes minimus*)	Skylark (*Alauda arvensis*)	**Goldfinch (*Carduelis carduelis*)**
Snipe (*Gallinago gallinago*)	Swallow (*Hirundo rustica*)	**Siskin (*Carduelis spinus*)**
Woodcock (*Scolopax rusticola*)	House martin (*Delichon urbicum*)	Linnet (*Carduelis cannabina*)
Curlew (*Numenius arquata*)	**Long-tailed tit (*Aegithalos caudatus*)**	Twite (*Carduelis flavirostris*)
Redshank (*Tringa totanus*)	Wood warbler (*Phylloscopus sibilatrix*)	Lesser redpoll (*Carduelis cabaret*)
**Black-headed gull (*Chroicocephalus ridibundus*)**	Chiffchaff (*Phylloscopus collybita*)	Common redpoll (*Carduelis flammea*)
Little gull (*Hydrocoloeus minutus*)	Willow warbler (*Phylloscopus trochilus*)	Common crossbill (*Loxia curvirostra*)
Mediterranean gull (*Larus melanocephalus*)	**Blackcap (*Sylvia atricapilla*)**	**Bullfinch (*Pyrrhula pyrrhula*)**
Common gull (*Larus canus*)	Garden warbler (*Sylvia borin)*	Hawfinch (*Coccothraustes coccothraustes*)
Lesser black-backed gull (*Larus fuscus*)	Lesser whitethroat (*Sylvia curruca*)	Snow bunting (*Plectrophenax nivalis*)
Herring gull (*Larus argentatus*)	Whitethroat (*Sylvia communis*)	Lapland bunting (*Calcarius lapponicus*)
Yellow-legged gull (*Larus michahellis*)	Waxwing (*Bombycilla garrulus*)	Yellowhammer (*Emberiza citrinella*)
Great black-backed gull (*Larus marinus*)	**Nuthatch (*Sitta europaea*)**	Cirl bunting (*Emberiza cirlus*)
**Feral pigeon (*Columba livia*)**	Treecreeper (*Certhia familiaris*)	Little bunting (*Emberiza pusilla*)
Stock dove (*Columba oenas*)	**Wren (*Troglodytes troglodytes*)**	**Reed bunting (*Emberiza schoeniclus*)**
**Woodpigeon (*Columba palumbus*)**	**Starling (*Sturnus vulgaris*)**	Corn bunting (*Emberiza calandra*)
**Collared dove (*Streptopelia decaocto*)**	Rose-coloured starling (*Pastor roseus*)	
Turtle dove (*Streptopelia turtur*)	Dipper (*Cinclus cinclus*)	

Supplementary Table 3| Change in winter feeder use between 1973 and 2012 for the 39 species defined as feeder-users.

Species	Scientific name	Direction of change	χ^2	*p**
Pheasant	*Phasianus colchicus*	+	162.89	<0.001 ***
Sparrowhawk	*Accipiter nisus*	+	405.85	<0.001 ***
Black-headed Gull	*Chroicocephalus ridibundus*		4.15	0.051 ·
Feral Pigeon	*Columba livia*	+	20.85	<0.001 ***
Woodpigeon	*Columba palumbus*	+	619.31	<0.001 ***
Collared Dove	*Streptopelia decaocto*	+	136.42	<0.001 ***
Great Spotted Woodpecker	*Dendrocopos major*	+	313.91	<0.001 ***
Magpie	*Pica pica*	+	228.62	<0.001 ***
Jay	*Garrulus glandarius*	+	43.58	<0.001 ***
Jackdaw	*Corvus monedula*	+	51.98	<0.001 ***
Rook	*Corvus frugilegus*		2.86	0.099 ·
Carrion Crow	*Corvus corone*	+	109.54	<0.001 ***
Goldcrest	*Regulus regulus*	+	11.46	0.001 ***
Blue Tit	*Cyanistes caeruleus*		3.63	0.067 ·
Great Tit	*Parus major*	+	6.19	0.017 *
Coal Tit	*Periparus ater*	+	22.63	<0.001 ***
Marsh Tit	*Poecile palustris*	-	54.08	<0.001 ***
Long-tailed Tit	*Aegithalos caudatus*	+	396.58	<0.001 ***
Blackcap	*Sylvia atricapilla*	+	110.57	<0.001 ***
Nuthatch	*Sitta europaea*	+	75.88	<0.001 ***
Wren	*Troglodytes troglodytes*	+	6.02	0.018 *
Starling	*Sturnus vulgaris*	-	85.26	<0.001 ***
Blackbird	*Turdus merula*		0.46	0.510
Fieldfare	*Turdus pilaris*	+	26.01	<0.001 ***
Song Thrush	*Turdus philomelos*	-	166.20	<0.001 ***
Redwing	*Turdus iliacus*	+	10.40	0.001 ***
Mistle Thrush	*Turdus viscivorus*	-	39.80	<0.001 ***
Robin	*Erithacus rubecula*	+	8.41	0.005 **
Dunnock	*Prunella modularis*		1.52	0.229
House Sparrow	*Passer domesticus*	-	71.50	<0.001 ***
Tree Sparrow	*Passer montanus*	+	11.18	0.001 ***
Pied Wagtail	*Motacilla alba*		3.57	0.068 ·
Chaffinch	*Fringilla coelebs*	+	24.82	<0.001 ***
Brambling	*Fringilla montifringilla*	+	197.51	<0.001 ***
Greenfinch	*Chloris chloris*		0.00	0.977
Goldfinch	*Carduelis carduelis*	+	779.54	<0.001 ***
Siskin	*Carduelis spinus*	+	176.55	<0.001 ***
Bullfinch	*Pyrrhula pyrrhula*	+	27.19	<0.001 ***
Reed Bunting	*Emberiza schoeniclus*		3.14	0.085 ·

'Feeder use' is defined as the proportion of gardens where each species used feeders. **p*-values have been adjusted using false discovery rate corrections to account for multiple testing across species[1]. Level of significance is denoted as · $p \le 0.1$, * $p \le 0.05$, ** $p \le 0.01$, *** $p \le 0.001$. Species are ordered taxonomically.

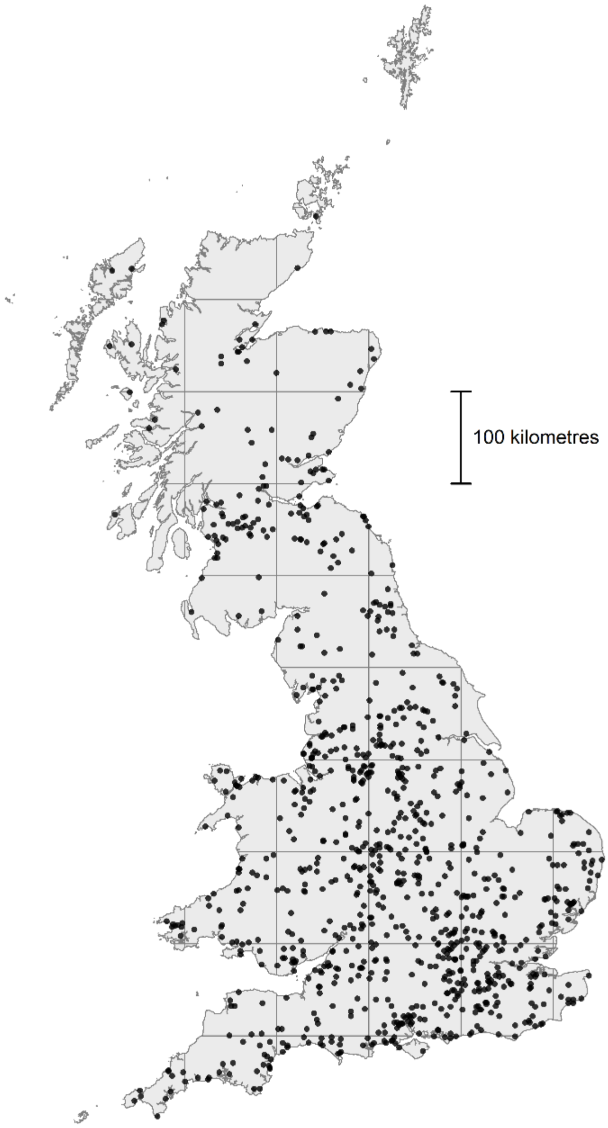

Supplementary Figure 1| Survey locations. The distribution of 1,001 gardens in Britain that were surveyed in at least one year as part of the Garden Bird Feeding Survey (GBFS) between the winters of 1973/74 and 2012/13. Map produced using R version 3.4.3, base map used with permission.

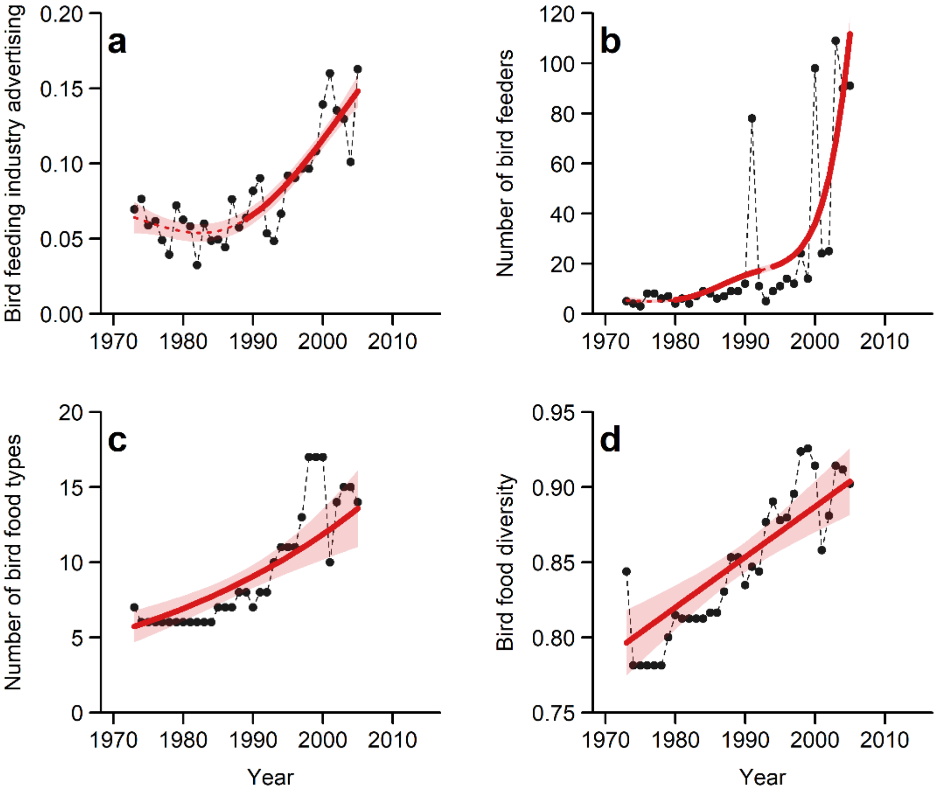

Supplementary Figure 2| Temporal changes in the British garden bird feeding industry. a-d trends in the proportion of advertising in RSPB *Birds* magazine filled by the garden bird feeding industry over 33 years, between 1973 and 2005, **(a)** the number of unique feeder products advertised **(b)**, the number of different food types advertised **(c)** and food diversity using Simpson's diversity index **(d).** Trends are shown with 95% confidence limits, periods of significant change are shown with a solid line and non-significant periods with a dashed line.

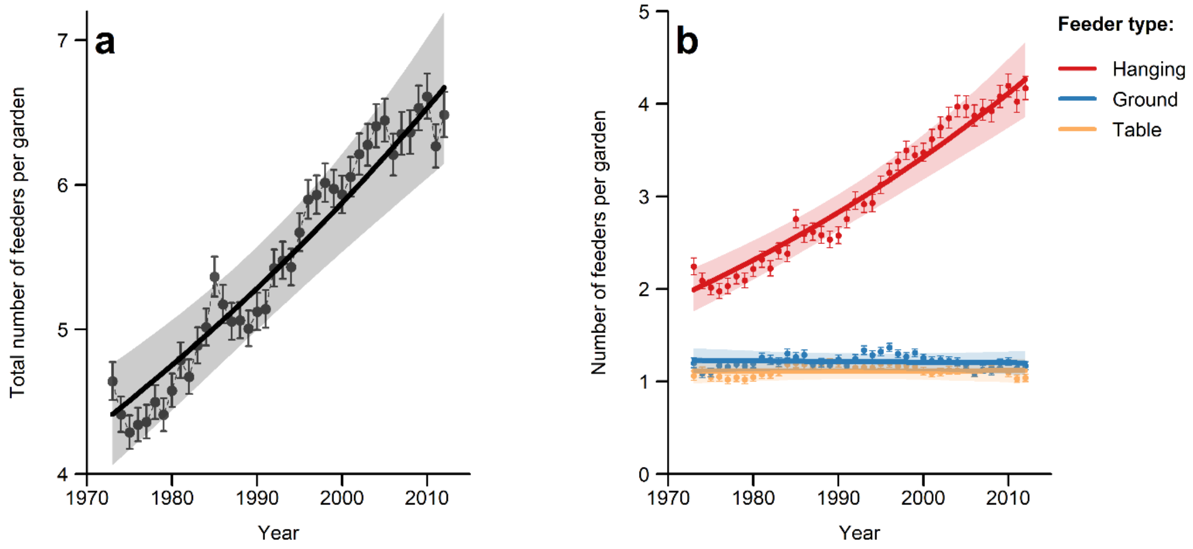

Supplementary Figure 3| Long-term changes in garden bird feeding practices. (a) trend in the mean total number of garden bird feeders provided in gardens each week in winter (n = 7433 garden-years). **(b)** Comparison of trends in the numbers of feeders when broken down by feeder type, using separate analyses for hanging (red), table (yellow) and ground (blue) feeders. Trends for table (p= 0.95) and ground (p= 0.58) feeders were not significant. Error bars around annual means depict s.e.m., lines are fitted using modelled predictions and the shading represents 95% confidence limits.

a

	Simpson's diversity		Species richness	
	t-value	*p*	*t*-value	*p*
smooth (Year)	171.46	<0.001	8.03	<0.001
Number of feeders	3.47	<0.001	16.84	<0.001
Winter temperature	0.78	0.438	18.70	<0.001
Habitat	3.53	<0.001	3.23	0.001

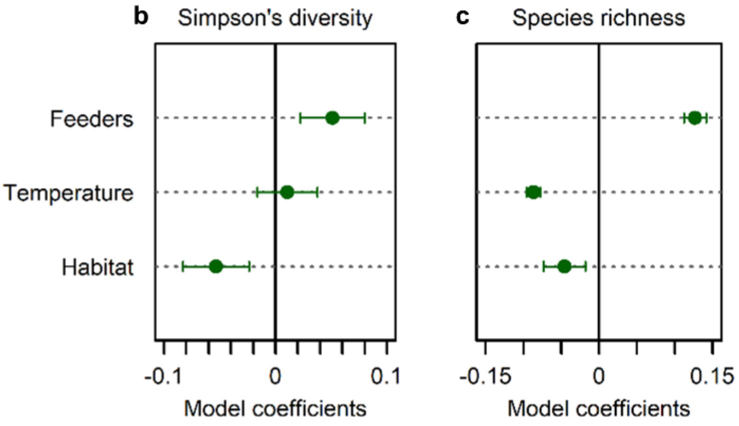

Supplementary Figure 4| Drivers of changing bird communities at garden feeders. (a) Results of GAMM models used to examine the drivers of variation in Simpson's diversity and species richness of birds using feeders in gardens (n = 7433 garden-years). **(b-c)** Standardised coefficients (± 95% confidence intervals) for the three linear terms shown in **(a)** when fitted against Simpson's diversity **(b)** and species richness **(c)**. The habitat coefficient describes suburban/urban gardens relative to rural gardens. Bars represent 95% confidence intervals around the estimates.

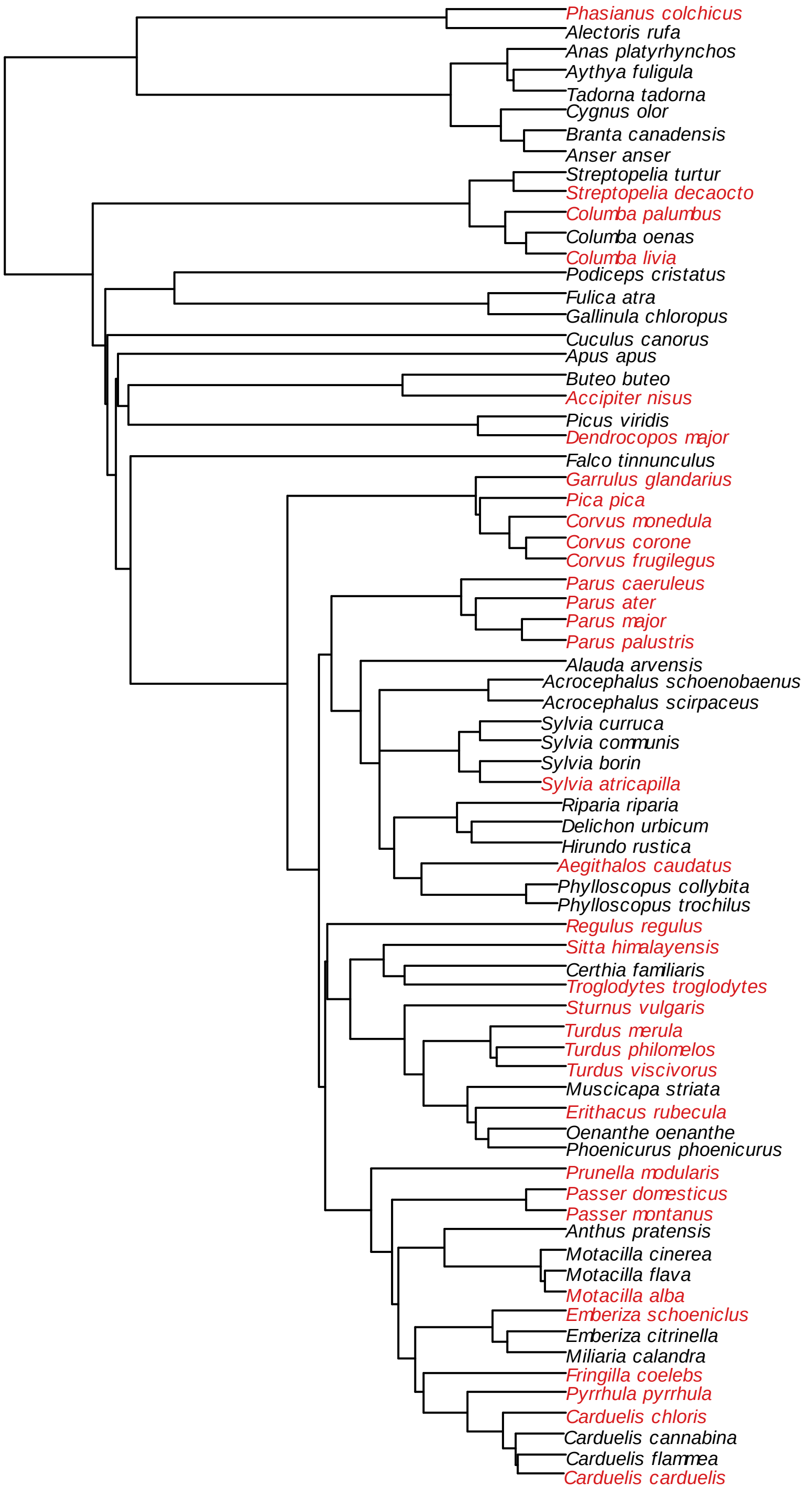

Supplementary Figure 5| Phylogeny of 73 bird species that do and do not use feeders. Consensus phylogenetic tree used when examining relationships between species' use of garden bird feeders and changes to their national populations. Species defined as feeder-users are coloured red. Data extracted from avian phylogenies developed by Jetz et al.[2] (http://birdtree.org). Since the Eurasian nuthatch (*Sitta europaea*) and lesser redpoll (*Carduelis cabaret*) were absent from the global avian tree, they have been represented by phylogenetically similar species which have the same relative relationships to all other species, the white-tailed nuthatch (*S. himalyensis*)[3] and the common redpoll (*C. flammea*)[4] respectively.

References

1 Pike, N. Using false discovery rates for multiple comparisons in ecology and evolution. *Methods Ecol Evol* **2**, 278-282 (2011).

2 Jetz, W., Thomas, G. H., Joy, J. B., Hartmann, K. & Mooers, A. O. The global diversity of birds in space and time. *Nature* **491**, 444-448 (2012).

3 Pasquet, E. Phylogeny of the nuthatches of the *Sitta canadensis* group and its evolutionary and biogeographic implications. *Ibis* **140**, 150-156 (1998).

4 Knox, A. G., Helbig, A. J., Parkin, D. T. & Sangster, G. The taxonomic status of Lesser Redpoll. *Br Birds* **94**, 260-267 (2001).
